# A Forward Phenotypically Driven Unbiased Genetic Analysis of Host Genes That Moderate Herpes Simplex Virus Virulence and Stromal Keratitis in Mice

**DOI:** 10.1371/journal.pone.0092342

**Published:** 2014-03-20

**Authors:** Richard L. Thompson, Robert W. Williams, Malak Kotb, Nancy M. Sawtell

**Affiliations:** 1 Department of Molecular Genetics, Microbiology, and Biochemistry, University of Cincinnati College of Medicine, Cincinnati, Ohio, United States of America; 2 Center of Genomics and Bioinformatics and Department of Anatomy and Neurobiology, University of Tennessee Health Science Center, Memphis, Tennessee, United States of America; 3 Division of Infectious Diseases, Cincinnati Children’s Hospital Medical Center, Cincinnati, Ohio, United States of America; University of Cambridge, United Kingdom

## Abstract

Both viral and host genetics affect the outcome of herpes simplex virus type 1 (HSV-1) infection in humans and experimental models. Little is known about specific host gene variants and molecular networks that influence herpetic disease progression, severity, and episodic reactivation. To identify such host gene variants we have initiated a forward genetic analysis using the expanded family of BXD strains, all derived from crosses between C57BL/6J and DBA/2J strains of mice. One parent is highly resistant and one highly susceptible to HSV-1. Both strains have also been fully sequenced, greatly facilitating the search for genetic modifiers that contribute to differences in HSV-1 infection. We monitored diverse disease phenotypes following infection with HSV-1 strain 17syn+ including percent mortality (herpes simplex encephalitis, HSE), body weight loss, severity of herpetic stromal keratitis (HSK), spleen weight, serum neutralizing antibody titers, and viral titers in tear films in BXD strains. A significant quantitative trait locus (QTL) on chromosome (Chr) 16 was found to associate with both percent mortality and HSK severity. Importantly, this QTL maps close to a human QTL and the gene proposed to be associated with the frequency of recurrent herpetic labialis (cold sores). This suggests that a single host locus may influence these seemingly diverse HSV-1 pathogenic phenotypes by as yet unknown mechanisms. Additional suggestive QTLs for percent mortality were identified—one on Chr X that is epistatically associated with that on Chr 16. As would be anticipated the Chr 16 QTL also modulated weight loss, reaching significance in females. A second significant QTL for maximum weight loss in male and female mice was mapped to Chr 12. To our knowledge this is the first report of a host genetic locus that modulates the severity of both herpetic disease in the nervous system and herpetic stromal keratitis.

## Introduction

Over 5 billion people are infected worldwide with herpes simplex virus type 1 (HSV-1) and will remain so for life constituting a reservoir of virus with the potential to infect new hosts. While many infections are asymptomatic, serious disease occurs in some individuals and HSV is the leading cause of both sporadic necrotizing encephalitis and infectious blindness in the United States [1]–[4]. Significantly, genital infection with HSV-2 is associated with a two-fold increased risk of transmission in sexually transmitted cases of HIV [5]–[7]. The ubiquity of HSV in the human population is the result of its ability to establish latent infections in sensory neurons and subsequently reactivate to cause recurrent disease and transmission to new hosts. No effective vaccine is yet available. Effective anti-HSV drugs exist, but resistant strains can develop [8]–[10], and antivirals cannot eliminate the latent viral reservoir. Advances in genomics present the opportunity to gain insight into host genetic variation that predispose to serious disease outcomes. This knowledge in turn can lead to enhanced treatment protocols, the design of new strategies to reduce latent reservoirs, and the potential to discover biomarkers to identify individuals at greater risk of severe herpetic disease.

About 40 years ago it was shown that both viral and host genetics contribute greatly to the outcome of experimental infection in mice [11], [12]. Other factors that impact HSV disease outcome in mice include the age of the host at the time of infection, the route of infection and the inoculation titer. Traditional reverse genetic approaches (e.g. gene knock out, knock down, or over expression strategies) have provided insight into the viral genes that regulate pathogenic processes and the role that the components of the host immune system plays in the formation of herpetic stromal keratitis [13]–[17]. However, little is known about naturally occurring host gene variants and molecular networks that modulate the infectious process or predispose the host to severe outcomes. A very early study suggested that a gene or genes residing close to the immunoglobulin heavy chain locus were involved in keratitis in mice [18]. Cantin and colleagues describe three loci on mouse chromosome (Chr) 6 involved in neutrophil function and resistance to fatal disease in mice [19]. Recently a human genetic locus associated with frequency of recurrent HSV-1 *labialis* (cold sores) has been mapped to Chr 21 [20], [21]. Despite these successes, there is much left to learn about the naturally occurring gene variants and molecular networks that contribute to protection against severe disease caused by herpes simplex virus.

We employed the BXD advanced recombinant inbred (ARI, [22]) lines of mice to identify novel gene variants that impact the severity of several important HSV-1 disease phenotypes including percent mortality (viral invasion of the central nervous system resulting in death) and herpetic stromal keratitis, in a well-characterized mouse model of HSV-1 corneal infection. The BXD gene reference population (GRP) is particularly well-suited for these studies for several reasons. First, the lines result from a cross of C57BL/6J (B6 or B) and DBA/2J (D2 or D) inbred strains of mice that are highly resistant and highly susceptible to HSV infection respectively [12]. In addition both parental strains have been deeply and completely sequenced and the BXD lines have been densely genotyped. Thus interval mapping for the identification of quantitative trait loci (QTL) is greatly expedited. The BXD lines have been used by researchers throughout the world in many diverse studies including those centered on the eye and nervous system (for example see [23]–[27]), as well as several infectious agents (for example [28]–[31]). The BXD family is one of the largest GRP, presently containing ∼160 lines of mice, each line of which is isogenic except for sex chromosomes. This large family permits the efficient identification of naturally occurring gene variants and molecular networks that regulate biologic properties. In addition a large number of previously published and as yet unpublished phenotypes and omics data sets on these strains can be accessed directly using the GeneNetwork.org site that also incorporates software for mapping and statistical analysis of the BXD and many other populations.

Here we infected the BXD lines and the parental strains with HSV-1 strain 17syn+ via the cornea and systematically phenotyped mice for >30 days post-infection. We report a novel QTL on Chr 16 that controls both virulence and HSK severity. This Chr 16 locus is also linked to maximum weight loss in females. A second QTL for maximum weight loss maps to Chr 12. The Chr 16 interval is homologous to human Chr 21, and the region identified here maps close to, but appears to be distinct from, the region on human Chr 21 that influences cold sore frequency in humans [20]. Our findings suggest that a single host locus may regulate both percent mortality and HSK severity in mice (and perhaps recurrent disease frequency in humans [20]) by some common as yet unidentified mechanism, which is discussed further below.

## Methods

### Ethics Statement

This study was carried out in complete accordance with the recommendations in the Guide for the Care and Use of Laboratory Animals of the National Institutes of Health. Animals were housed in American Association for Laboratory Animal Care-approved quarters. The protocol (internal protocol number 2E06052) was approved by the Animal Care and Use Committee of the Cincinnati Children’s Hospital Medical Center (PHS assurance# - A3108-01). No surgical procedures were performed.

### Infection and phenotyping

Herpes simplex virus type 1 (HSV-1) infection: HSV-1 strain 17syn+ was propagated on cultured rabbit skin cells (RSC, originally obtained from Bernard Roizman at the University of Chicago) and viral titers were determined by serial-dilution plaque assay as previously detailed [32]–[34]. The wild type HSV-1 laboratory strain 17syn+ was originally obtained from John H. Subak-Sharpe at the MRC Virology Unit in Glasgow, Scotland. Animals were housed in American Association for Laboratory Animal Care approved quarters. Male and female C57BL/6J and DBA/2J mice (4 to 6 weeks old) were obtained from Jackson Laboratories. At least four male and four female mice (4 to 6 weeks old) of the various BXD strains were employed and most strains were analyzed more than once throughout the multi-year study. BXD mice were obtained from the colony at the University of Tennessee Health Science Center or a colony maintained at the University of Cincinnati.

Prior to inoculation, mice were anesthetized by intraperitoneal injection of sodium pentobarbital (50 mg/kg of body weight). A 10 μl drop containing 1×10^6^ pfu of HSV strain 17syn+ was placed onto each scarified corneal surface as previously detailed [32]–[34]. Virus titers in tear films were assayed on day 4 p.i. Mice were observed daily and weighed every other day until weight returned to that recorded prior to infection. Mice were euthanized if 30% of starting body weight was lost. Percent mortality was scored from 48 hours through 21 days p.i. Any deaths occurring outside of this range were not attributed to acute viral infection. Eyes were examined by two independent observers and scored on a 0 to 5 point scale: 0 =  no visible corneal opacity; 1 =  up to 25% cornea involvement; 2 =  25–50% cornea involvement; 3 =  50–75% cornea involvement; 4 =  75–100% cornea involvement; and 5 =  penetrating keratitis, essentially as previously detailed [35]–[37]. At 30 to 35 days p.i., mice were necropsied, major organs and draining lymph nodes were examined grossly, spleen weights recorded, and blood samples taken for serum virus neutralizing antibody titer analysis. Eyes were routinely processed for histological examination. Following overnight fixation in 4% paraformaldehyde, eyes were embedded in paraffin, sectioned and stained with cresyl violet for histological examination of the cornea.

### Genome wide data analysis

QTL mapping. Quantitative trait locus (QTL) mapping was performed with the GeneNetwork analysis tools (www.genenetwork.org). Single marker regression was performed across the entire mouse complement of chromosomes at markers typed across BXD strains. A likelihood ratio statistic (LRS) was calculated at each marker comparing the hypothesis that the marker is associated with the phenotype with the null hypothesis that there is no association between marker and phenotype. Genome-wide significance was determined by performing 2000 permutations. Significant QTLs were found on Chr 12 and 16 (weight loss), Chr X and 16 (percent mortality) and Chr 16 (herpetic stromal keratitis severity). Other tools available at www.genenetwork.org were employed for pair-wise scans and Spearman rank correlation statistical analyses.

## Results

Thirty-two fully inbred BXD strains were phenotyped. Groups of each line, including males and females in approximately equal numbers, were infected with wild type HSV-1 strain 17syn+ on the cornea as detailed in Methods. A single investigator infected all of the mice to ensure high technical consistency. Infected mice were observed daily, weighed every other day, and tear films were collected on day 4 post-infection (p.i.) to evaluate viral titers. Eyes were independently scored by two investigators for the severity of herpetic stromal keratitis essentially as previously described [35], [36], and as detailed further below and in methods. The parental strains and most BXD lines were analyzed more than once throughout the multi-year study in groups ranging from 3–10. In all cases the reproducibility of the disease phenotypes displayed within individual lines was consistent. In contrast, extensive variability in the phenotypes was displayed between lines.

### General patterns of disease

Clinical and postmortem findings in humans combined with animal model studies provide a temporal and spatial understanding of primary HSV infection. In the mouse corneal infection model with viral strains of moderate virulence, viral replication occurs in an overlapping wave in three distinct tissue compartments including corneal epithelium, innervating trigeminal ganglion (TG), and connecting central nervous system (CNS). This acute phase of primary infection resolves in 8–12 days. Viral replication on the cornea peaks at day 4 p.i. By 2 days p.i. virus that has transported through sensory neuron axons to the TG begins to replicate and viral titers reach their peak on day 4 p.i. in TG, and infectious virus is cleared within 8 to 9 days p.i. During this time virus enters the CNS, and it is the timing and extent of viral replication here that determines whether or not lethal encephalitis ensues [33], [38]–[42].

**Figure 1:**
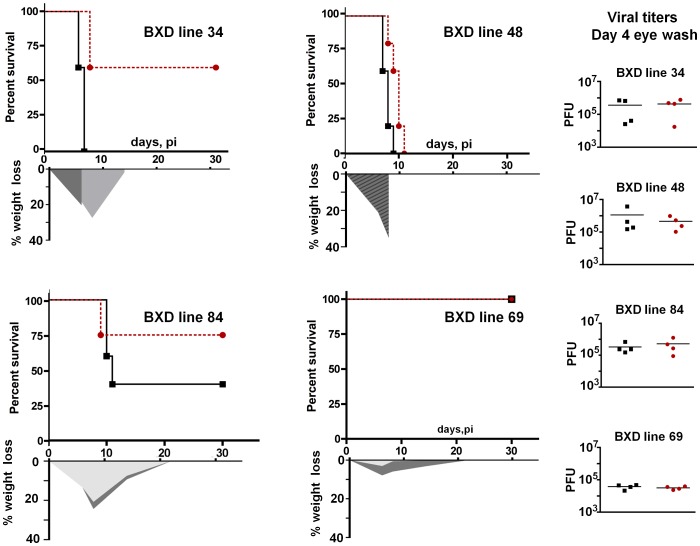
Representitive results with several BXD strains. Shown are representitive data from four different BXD lines. The top Y axis in each panel is the percent survival and the bottom Y axis is the percent weight loss. The X axis is the time in Days post infection (p.i.). The scattergrams on the right show the tear film titers of individual mouse eyes at 4 days p.i. Red circles and dashed lines  =  female; Black squares and solid lines =  male. In the experiment shown, which is a small subset of the data generated, four male and four female mice of each BXD strain were employed.

With respect to susceptibility to fatal encephalitis, four general patterns were observed (fig. 1). Some of the BXD lines were highly resistant as is the B6 parent strain. This outcome is typified by BXD69. In this line, 100% of both males and females survived infection with minimal weight loss. At its maximum, less than 10% of original body weight was lost which had returned to preinfection values within 12 days p.i. In contrast, other lines were highly susceptible with no survivors, similar to the D2 parent at the inoculation titer employed. The BXD48 line typifies the response of these susceptible lines. Both males and females of this line continuously lost weight and succumbed to infection by day 12 p.i.

In some lines, males and females exhibited susceptibility differences as typified in BXD34 strain (fig.1). This sex bias in mouse susceptibility to HSV infection is a recognized phenomenon and there is some suggestion that the same bias exists in humans [43], [44]. In general, mice in these groups lost about 25% of their body weight within days 8 p.i. In those lines surviving, body weight was recovered over the next 10 days. We noted that males in these groups lost less weight at the time of death than surviving females in the same group. Viral replication on the eye was similar for both males and females in these groups (fig. 1 and data not shown). A sex difference in susceptibility was not universal, however, as many lines exhibited a similar percent mortality in males and females (fig. 1, e.g. BXD69, BXD48), indicating that host genetic loci can moderate the effect of sex.

#### Interval mapping and identification of significant QTL

Gene mapping methods systematically evaluate the statistical significance of linkage between genetic markers (e.g. SNPs or microsatellites) across the whole genome and differences in phenotypes. We performed mapping using the GeneNetwork. Interval maps are computed using the Haley-Knott regression method (for review see [45]). Permutation analysis was used to estimate the empirical genome-wide p values associated with a given likelihood ratio statistic (LRS) score. The LRS is mathematically related to the log of odds (LOD) ratio that is often employed in genetic analyses and LRS can be converted to LOD by dividing the LRS score by 4.61.

#### Genome wide linkage scan interval mapping for maximum weight loss

**Figure 2:**
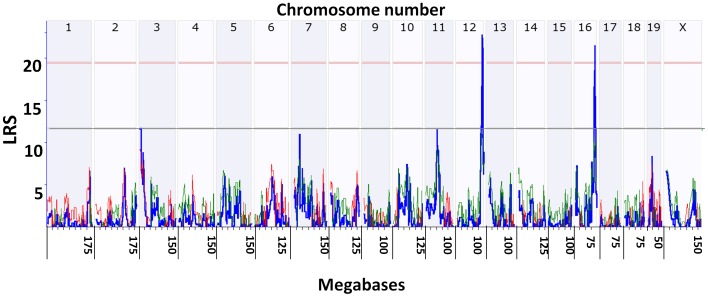
Quantitative trait loci for percent maximum weight loss. Mice were infected as described in methods and weighed every other day. Analysis tools in genenetwork (GN) available at www.genenetwork.org were employed to search for possible quantitative trait loci (QTL) in male and female mice. The maximum percent weight loss was employed to search for genome wide association. Shown is a screen capture from GN for female mice. The X axis shows the mouse chromosomes from chromosome 1 to X from the centromere to the end (mouse chromosomes have only one arm). The long ticks are at base pair number 1 of each chromosome and the short ticks are every 25 megabases. The Y axis is the likelihood ratio statistic (LRS) score. 2000 permutation tests were employed to estimate the genome wide adjusted suggestive LRS (p = 0.37, grey horizontal line) and the genome wide adjusted significant LRS (p = 0.05, red horizontal line). The wavy blue line indicates the local LRS at various SNPs etc. across the entire mouse genome. The colored lines show the additive effect of the influence of the locus (red lines indicate association of DBA/2J with trait values, green lines indicate that C57BL/6J alleles increase trait values). Significant QTL for percent maximum weight loss were mapped to Chr12 for both male and female mice and on Chr16 for female mice (shown). The QTL on 16 was suggestive in male mice (not shown). A fully functional interactive analysis and graphic interface available to the public can be generated on GN for trait ID:16194 as detailed in methods. On GN the user can restrict the output to the specific chromosomal region under the peak of the QTL and explore the genes, single nucleotide polymorphisms (SNPs) etc. present in the location (for example see fig. 7).

Weight loss is an effective measure of infectious disease severity. We detected significant QTL for percent maximum weight loss on Chr 12 and 16 in female mice (see fig. 2) and on Chr 12 in male mice, which also had a suggestive QTL on Chr 16 (not shown). Figure 2 is a screen shot of an interactive graphic interface in GN and shows the result of a genome wide analysis of percent weight loss in female mice. The peak LRS was 22 on Chr 12 at ∼114.5 Mb. We detected a second significant QTL on Chr 16 in female mice with an LRS of 21 at ∼89.4 Mb. This region of Chr 12 was previously associated with keratitis by analysis of congenic mouse strains [18]. In contrast, the Chr 16 interval has not been identified previously for HSV disease resistance. The results of a pair scan analysis that tests for all possible two-locus epistatic interactions did not suggest any interaction between the loci on Chr 12 and 16. Note that the original data consisting of 11 herpes-associated phenotypes generated as part of this study, along with high resolution, dynamic, and interactive plots and maps can be easily regenerated by searching GeneNetwork for *Species*  =  Mouse, *Group*  =  BXD, *Type*  =  Phenotypes, and then entering the search string “herpes”.

#### Genome wide linkage scan interval mapping for percent mortality

**Figure 3:**
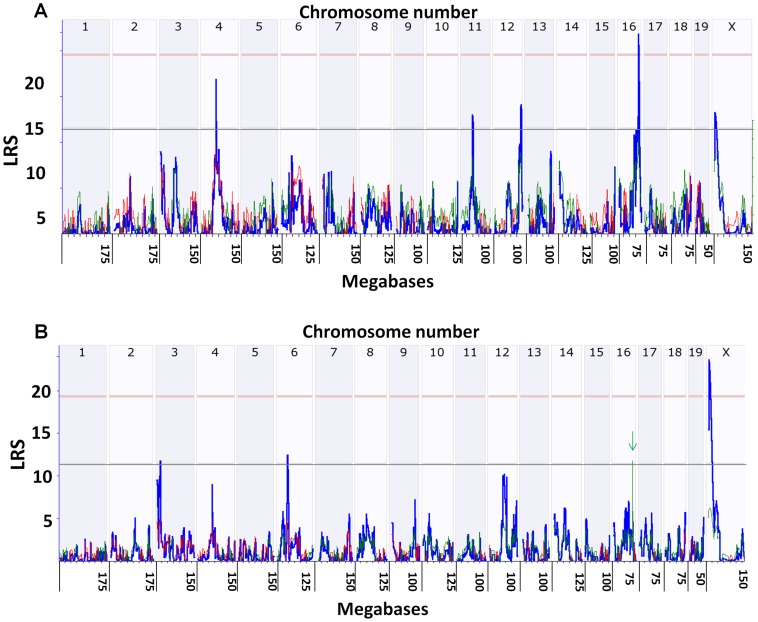
Quantitative trait loci for percent survival. Percent survival of male and female mice combined was employed to search for QTL associated with mortality. See the legend for fig. 2 for an explanation of the lines in the figure. A significant QTL was mapped to the distal end of Chr 16 and suggestive QTL were localized to Chr 4, 11, 12 and X (panel A). A pair scan analysis revealed a possible interaction between the locus on 16 and the suggestive locus on X (not shown). The marker regression function was used to identify SNPs with high LRS scores on Chr 16 and X, and the composite scan analysis was then employed to control for the Chr 16 or the Chr X locus. Controlling for the QTL on 16 (green arrow and line) revealed that the suggestive QTL on X was significant (panel B).

We also detected a significant QTL associated with percent mortality on the distal portion of chromosome 16 (maximum LRS  =  29 at 86.2 Mb, fig. 3). Infected mice of susceptible strains showed signs of central nervous system disease including hunched posture, roughened fur, convulsions and/or ataxia, indicating that death was the result of herpetic encephalitis. Several suggestive QTL were found on Chrs 4, 11, 12, and X. The strongest of these suggestive QTL with an LRS of 17.5 was on Chr 4. Of interest, the susceptibility allele at the Chr 4 locus is inherited from the resistant B6 parent (red line in fig. 3). A pair scan analysis suggested a possible interaction between Chr 16 and the suggestive QTL on Chr X (not shown). We performed composite mapping to control for possible masking effects of the QTL on Chr 16. Epistasis, the moderation of the phenotypic effect of alleles at one gene by alleles of another gene, can sometimes be revealed by this approach and secondary, but still significant loci can be identified. We detected a significant locus on the proximal part of Chr X with a peak LRS of 24 when we controlled for SNP rs4213268 on Chr 16 (fig. 3B bottom panel, green arrow and line).

#### Genome wide linkage scan interval mapping for herpetic stromal keratitis

**Figure 4:**
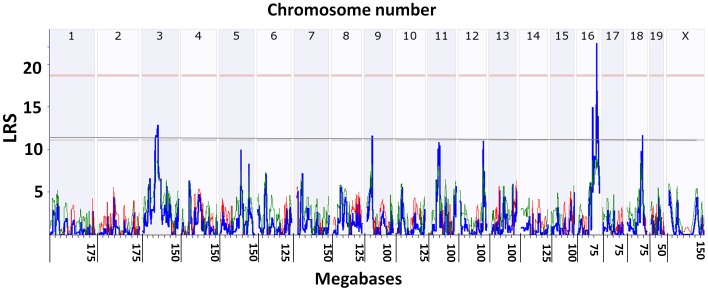
Quantitative trait loci associated with herpetic stromal keratitis. BXD strains of mice were infected via the cornea and the severity of stromal keratitis in male and female mice combined determined by two independent observers using a 5 point scale (0 =  no disease, 5 =  penetrating stromal keratitis). Analysis tools in genenetwork available at www.genenetwork.org were employed to search for possible quantitative trait loci (QTL). See the legend for fig. 2 for an explanation of the lines in the figure. A significant quantitative trait locus (QTL) for severity of herpetic stromal keratitis was identified on Ch 16. A fully functional interactive analysis and graphic interface is available on GN for trait ID:16186.

Two independent investigators scored the severity of herpetic stromal keratitis throughout the acute stage of infection, and final scores were obtained on day 30 p.i. A scale 0 to 5 was based on previously published criteria [35]–[37], [46] as detailed in methods and also by histochemical analysis of corneal tissues. The average score of all the eyes in a group ± standard error was employed for interval mapping. A statistically significant QTL for HSK severity was localized to the same region of the distal arm of Chr 16 as was significant for percent mortality. The peak LRS for virulence mapped to 86.45 Mb (fig. 3A) and the peak LRS for HSK mapped to 86.16 Mb (fig. 4). Suggestive QTL were also observed on Chrs 3, 8, 11, 12, and 18. Controlling for the QTL on Chr 16 did not reveal any additional significant QTL. Likewise pair scans did not detect interactions.

**Figure 5:**
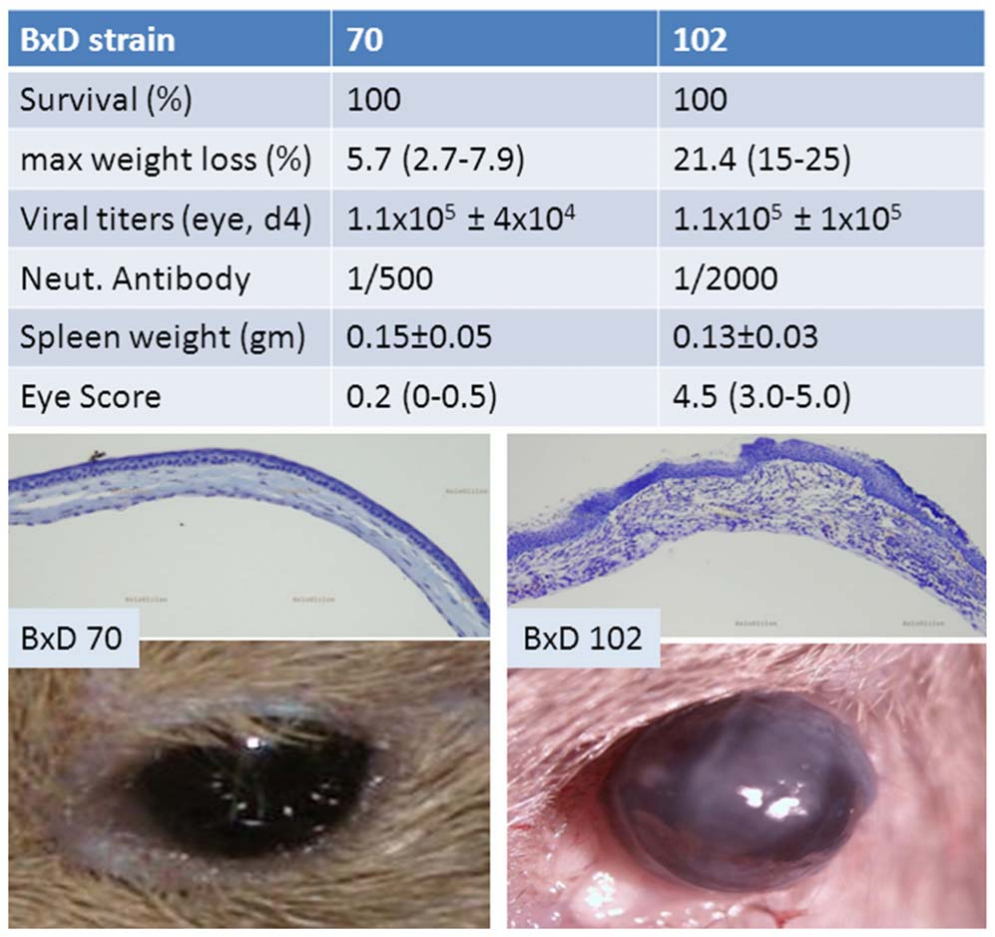
Representitive data in BXD lines that differ at the HSK QTL on Chr 16. Groups of male and female mice were infected as described in methods with 1×10^6^ pfu of HSV-1 strain 17syn+. The indicated parameters were assayed as described in the text and eyes were scored at 30 days p.i. by two independent investigators. The top micrographs are of cresyl violet stained corneal sections processed at 30 days p.i. Deranged and thickened corneal epithelium, neovascularization, derangned stromal keratocytes and greatly thickened stromal layer with immune cellular infiltrate is seen in the BXD102 strain. The BXD70 cornea appears normal. The bottom photos are of representitive live mouse eyes 30 days p.i. The BXD70 mouse eye appears normal. Neovascularization and stromal opacity covering more than 75% of the cornea is seen in the BXD102 eye.

Representative photographs of whole eyes and photomicrographs of sectioned corneas are shown in fig. 5. As seen in the photographs of live animals at 30 days p.i., eyes of BXD70 appear normal, whereas those of BXD102 have significant opacity covering most of the cornea. Microscopic examination of the corneas extended these findings. Corneas from BXD70 appear normal, whereas thickening and derangement of the epithelial and stromal layers, and neovascularization of the stromal layer is pervasive in BXD102 corneas (fig. 5.).

The BXD70 and BXD102 differ at the HSK locus on 16 QTL, with line BXD70 having the C57BL/6J haplotype and BXD102 having the DBA/2J haplotype across the entire region. This region co-maps with percent mortality, although these particular lines had 100 percent survival of both males and females. Weight loss, a general measure of disease severity, was significantly greater in BXD102 mice (p ≤ 0.05, Student *t* test). BXD70 mice had very little eye disease with an average score of 0.2 compared to an average score of 4.5 for BXD102.

**Figure 6:**
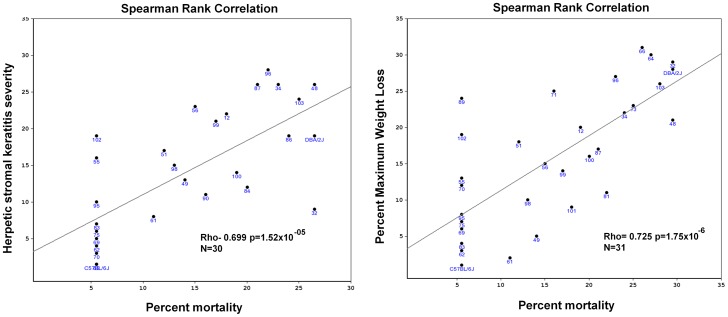
Correlations between phenotypes that map to the distal end of Chr 16. Spearman Rank correlations were performed bewteen percent mortality and herpetic stromal keratitis datasets (left panel, p = 1.52×10^−05^) or percent maximum weight loss of both male and female mice combined and percent mortality of male and female mice combined (right panel, p =  1.75×10^−06^).

In general, those BXD strains that exhibited more severe disease signs or greater mortality had higher serum antibody neutralization titers. A QTL for serum neutralization titer was not obtained, and this may be due at least in part to the fact that many and in some cases all of the animals of a susceptible BXD strain succumbed to infection before serums were obtained at 30 days p.i. Strains in which sera from at least three survivors was obtained were included in the analysis. Since the phenotypes of percent mortality, percent maximum weight loss and HSK had QTL that mapped to Chr 16, we performed Spearman rank correlations to determine if the co-mapping was statistically significant. The Spearman rank correlation is a non-parametric measure that assesses how well the relationship between two variables can be described using a monotonic function. We found that percent mortality was significantly correlated to both weight loss and HSK severity, with the former being the stronger association (fig. 6).

## Discussion

Translating the advances in genomic technology into progress toward realizing the broad benefits promised by individualized medicine will require protocols to determine each individual’s susceptibility to disease states and sensitivity to treatment and prevention regimens. Analysis of genetic reference populations (GRP) coupled with genome wide association studies (GWAS) can lead to the identification of naturally occurring gene variants associated with severe disease outcomes and deduction of the gene networks to which they contribute. This information in turn can identify biomarkers of susceptibility and also suggest potential new treatment strategies. Herpes simplex virus type 1 (HSV-1) infection is an example of an infectious disease which is normally relatively benign in most individuals but can cause severe disease sequelae in otherwise healthy people. There is at present no way to predict who might be susceptible to these rare severe manifestations of herpetic disease.

Our current understanding of HSV-1 disease mechanisms comes largely from reverse genetics approaches such as gene deletions in mice and the virus or RNAi knock downs of viral or host genes. A large body of literature defines the importance of many viral genes and host immune modulating factors, cellular infiltrates, cytokines and angiogenic factors, and potential autoimmunity in viral virulence and/or the formation of the corneal scaring and loss of visual acuity (reviewed in [13], [16], [36], [47]–[50]. However, these studies do not identify those individuals potentially at high risk. We employed an unbiased forward phenotypically driven systems genetics approach to begin to identify unknown gene variants and potential molecular networks of the host that contribute to these severe herpetic disease states, with the ultimate goals of disease risk prediction and improved treatment outcomes. It is of interest that none of the aforementioned host genes or molecular mechanisms was directly implicated in the QTLs we identified.

In this study a single well-characterized HSV-1 laboratory isolate (17syn+) and a single inoculation titer were employed in the BXD family using a generally well characterized model of ocular HSV infection. The BXDs are particularly well suited for these studies because the parental strains are either very resistant to HSV-1 (C57BL/6J) or very sensitive (DBA/2J). The BXD lines have the advantage of being widely available and they have a 40-year history of use [51]. The BXDs have also been thoroughly genotyped and phenotyped and many large omics data sets generated in prior studies can be mined to, for example, identify relevant pleiotropic interactions and expression QTL (eQTL) in many tissues and cell types.

Our analysis revealed and mapped significant QTLs on Chr12 and 16 (weight loss), Chr X and 16 (percent mortality) and Chr 16 (herpetic stromal keratitis severity). Somewhat surprisingly a common QTL for all three of these phenotypes overlaps on the distal end of Chr 16. Virulence of a given virus strain and its ability to cause HSK are not linked [35], [36]. Note however, that in one study, passage of viral isolates in vivo co-selected for increased neuroinvasiveness and increased HSK severity [35], [36]. A major common host QTL for both percent mortality (neuroinvasion) and HSK, while not predictable, may have important implications for how the process leading to HSK is initiated. This is currently under investigation.

There is a paucity of forward genetic analyses of HSV disease. Prior studies in mice implicated a region on Chr 6 that confers resistance to HSV infection that appears to be an autosomal dominant but is nevertheless strongly sex-biased [19]. Our studies did not detect this locus, presumably because the underlying variants are not segregating in the BXDs. Analysis of the eight parent Collaborative Cross mouse lines, when they become widely available, would cover more than 90% of mouse genetic diversity. An early study employed congenic lines of mice that varied at the immunoglobin heavy chain (IgH) locus and found an association with the locus on Chr 12 and keratitis [18]. A study performed on 129/SVEV x C57BL/6)F(2) mice at the ten centimorgan (cM) level revealed loci on Chr 3, 5, 12, 13 and 14 with impacts on general disease, and 10 and 17 associated with keratitis in complex with other loci [52]. Our study uncovered a highly significant QTL on Chr 12 locus for percent maximum weight loss. The QTL on the distal end of Chr 12 (106–119 Mb) contains the Igh locus, and this same region reached the suggestive linkage criterion for percent mortality and HSK severity. This region contains over 200 genes and further studies will be required to reduce this number of positional candidates to a manageable number.

While it is possible that IgH family members are involved in resistance to HSV, there was no direct correlation between serum neutralizing antibody titers in BXDs and percent mortality. A mechanism of molecular mimicry of an IgH locus and viral antigen leading to HSK has been described [47], but is limited to specific viral and mouse strains and dominant only under conditions of low viral load [53], which were not duplicated in our studies. Other potentially interesting genes within this region include YinYang1 (YY1), a transcription factor that can both up or down regulate genes through its cognate binding sites and is known to regulate HSV-1 genes [54], [55]. The YY1 gene contains a non-synonymous SNP between B6 and D2 and also has a cis expression QTL (cis-eQTL) at 110.06 Mb (determined with the Hippocampus Consortium data set in GN [56]), which regulates levels of expression in the nervous system. Such cis-eQTL are often implicated in disease phenotypes. This region of Chr 12 also contains the *Rest* gene. REST and co-REST proteins are thought to help silence the viral genome in cultured cells, and there is evidence this occurs in sensory neurons in vivo [57], [58]. The *Rest* gene has a cis-eQTL at ∼112.35 Mb.

A few forward genetic analyses of HSV disease severity in humans have been reported. In a series of phenotypically driven GWAS studies in highly inbred human populations, Cassanova and colleagues have demonstrated the importance of the innate immune response in protection. In particular, they found TOLL receptor signaling/interferon gamma pathways important for protection of the central nervous system from HSV-1 encephalitis. Rare individuals with defects in these pathways can suffer repeated bouts of HSE [59]–[61]. However the vast majority of people who experience HSE do not have frank defects in these pathways and there is as yet no evidence that certain alleles predispose to greater risk. These studies are akin to and confirm prior reverse genetic studies performed in mouse models in that the defects ablated gene function and emphasize the importance of the innate interferon response pathways in preventing serious CNS infection [62]–[66].

**Figure 7:**
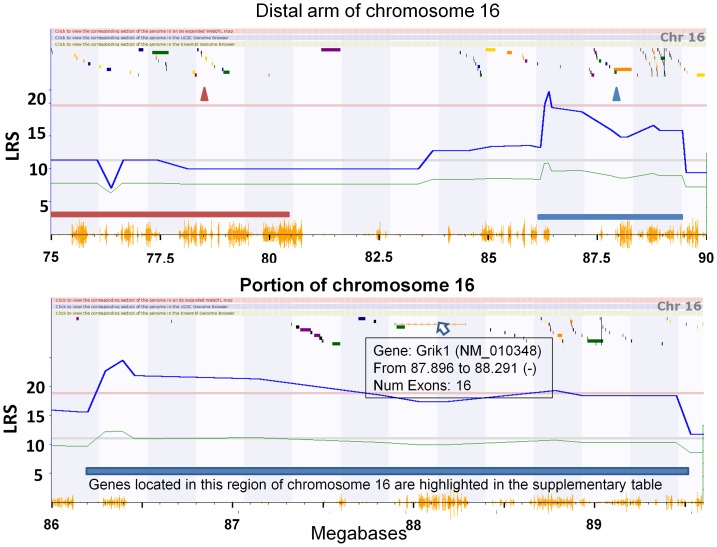
Schematic representation of the region of mouse Chr 16 from 75 to 90 mb. Suggestive (grey horizontal line) and significant (red horizontal line) LRS for HSV virulence (HSE) in male and female mice combined are indicated. The wavy blue line is the local LRS. The gold seismic graph along the X axis indicates local SNP density. The mouse susceptability QTL region is deliniated by a solid blue rectangle just above the SNP seismic graph. The region equivalent to the human cold sore frequency QTL is designated by a solid red rectangle just above the seismic graph at 75–82 mb. The location of the mouse equivalent (D16Ertd472e ) to the human “cold sore” gene (C21orf91) is indicated by a red triangle. A blue triangle indicates the 3’ end of the Grik1 gene that is an expression QTL for Grik1 in the nervous system. C21orf91 is a short gene shown as a gold box, which is not clearly discernable at this magnification (Top panel). Bottom panel. A three megabase region comprising the peak of the QTL is shown. Grik1, which is 395 kb in length is also shown as a gold rectangle and the other variously colored shapes indicate known or predicted genes and transcripts in the region. In GN, hovering the mouse cursor over the colored shapes (indicated by the open arrow in fig. 7, bottom) gives brief information about each gene. Clicking on the shapes calls up all related entries in the NCBI Gene database. The bands along the top are clickable and either increase the magnification, go to the respective region of the UCSC genome browser, or go to the respective region of the Ensemble genome browser. A fully functional and interactive figure can be generated and further explored on genenetwork.org for trait 16185 by performing the interval mapping function for Chr16.

A forward approach in a genetically more diverse human GRP examined frequency of herpes labialis (cold sores) and mapped frequent recurrent disease to a locus on human Chr 21, which is homologous to part of mouse Chr 16. The putative “cold sore” gene (*C21orf91*) which encodes a protein of unknown function, maps near to but is distinct from the QTL we have identified for percent mortality (HSE) and stromal keratitis severity (HSK, see fig. 7). A putative mouse gene highly homologous to *C21orf91* (also designated *D16Ertd472e*, *EURL*, *YG91*, and *CSSG1*) resides at the expected location of 78.54 Mb on mouse Chr 16 and there is one nonsynonymous SNP between the parental mouse strains. However, this gene does not overlap the murine QTL (fig. 7 top panel). There are at least two possible explanations for this result. The same gene may be involved in neuroinvasiveness/ HSK in mice and cold sore frequency in humans, and the studies map it to different locations due to mapping resolution. Alternately, distinct HSV resistance loci and genes may exist in this region of human Chr 21 and mouse Chr 16.

We examined the regions under and near the Chr 16 locus to identify candidate genes that might affect these phenotypic properties. The list was filtered down to a list of 63 candidates using factors such as the LRS value (fig. 7 bottom panel), known gene functions, known transcription levels in neural tissues and eyes (from Affymetrix and Illumina array and RNAseq databases in Genenetwork.org) as well as the density of SNPs (see Table S1). The 45 genes distal to 88.6 Mb are part of a keratin protein cluster [67] and are not likely to be involved. Of the remaining 18 genes under the locus only one—*Grik1*— is known to be expressed in sensory neurons [68]. *Grik1* encodes the GluR5 subunit of the kainate 1 ionotropic glutamate receptor that is detected in more than half of the neurons in dorsal root ganglia [68]. In addition *Grik1* mRNA levels in the nervous system are controlled by an expression QTL (eQTL) at 87.9 Mb and there is a very high SNP density within and around *Grik1* (fig 7). For these reasons *Grik1* is a top candidate gene for regulation of percent mortality (neuroinvasion) and severity of stromal keratitis. GluR5 is a ligand (glutamate) binding component of a kainate receptor that is involved in a large array of nervous system phenotypes such as complex behaviors and cocaine addiction (reviewed in [69]) including some previously associated with HSV infection such as schizophrenia [70], [71]. Other possible candidates include *Bach1*, a transcription factor thought to be ubiquitously expressed and implicated in spinal injury repair [72]. Listerin E3 ubiquitin ligase 1 (*Ltn1*, aka *Zfp294*) is expressed developmentally in neurons of the PNS and CNS [73]. These latter genes are somewhat less promising candidates than *Grik1* because they are associated with few if any C57BL/6J versus DBA/2J non-synonymous SNPs, indels, or cis-eQTLs.

Although the precise sequence variants involved in disease susceptibility to HSV-1 remain to be identified, the fact that host functions that regulate disease severity in mice (HSE and HSK, this study) and frequency of recurrent disease in humans [20], [74] map closely together implies there may be a previously unknown association between viral interaction with the nervous system and the severity of HSK. One potential mechanism is a host function that regulates the efficiency of the virus to transport in axons in the anterograde direction from neuron cell bodies in the trigeminal ganglia to the brain (i.e. percent mortality) or cornea (HSK severity) and possibly to the mucocutaneous region around the mouth in humans (cold sore frequency, [20]). Further studies may clarify whether the locus identified on mouse Chr 16 and/or the human Chr 21 is associated with an increased risk of HSK in humans. Finally, our studies have revealed BXD lines relatively resistant to HSE that are either highly susceptible, or highly resistant to the development of HSK. By analyzing the infectious disease dynamics in these selected strains using powerful omics approaches, it should be practical now to identify molecular networks and biomarkers associated with high risk for the formation of severe HSK.

## Supporting Information

Table S1 **Shown are the known and predicted genes under and near the Chr 16 QTL for percent mortality detailed in** **figure 7** **.** Genes within the QTL are highlited in light blue (entries 7 to 70). An interactive table containing more information can be generated at www.genenetwork.org by using the GeneNetwork Interval Analyst for Chr 16 gated between 85 and 90 Mb.
(DOCX)

## References

[1] Levitz RE (1998) Herpes simplex encephalitis: a review. Heart Lung 27: 209–212. 9622408 10.1016/s0147-9563(98)90009-7

[2] Whitley RJ (1990) Viral encephalitis. N Engl J Med 323: 242–250. 2195341 10.1056/NEJM199007263230406

[3] Whitley RJ (2002) Herpes simplex virus infection. Semin Pediatr Infect Dis 13: 6–11. 12118847 10.1053/spid.2002.29752

[4] Smith G (2012) Herpesvirus transport to the nervous system and back again. Annu Rev Microbiol 66: 153–176. 22726218 10.1146/annurev-micro-092611-150051 PMC3882149

[5] Corey L (2007) Synergistic copathogens—HIV-1 and HSV-2. N Engl J Med 356: 854–856. 17314346 10.1056/NEJMe068302

[6] Corey L, Wald A, Celum CL, Quinn TC (2004) The effects of herpes simplex virus-2 on HIV-1 acquisition and transmission: a review of two overlapping epidemics. J Acquir Immune Defic Syndr 35: 435–445. 15021308 10.1097/00126334-200404150-00001

[7] Glynn JR, Biraro S, Weiss HA (2009) Herpes simplex virus type 2: a key role in HIV incidence. Aids 23: 1595–1598. 19512858 10.1097/QAD.0b013e32832e15e8

[8] Horsburgh BC, Chen SH, Hu A, Mulamba GB, Burns WH, et al (1998) Recurrent acyclovir-resistant herpes simplex in an immunocompromised patient: can strain differences compensate for loss of thymidine kinase in pathogenesis? J Infect Dis 178: 618–625. 9728528 10.1086/515375

[9] Griffiths A, Chen SH, Horsburgh BC, Coen DM (2003) Translational compensation of a frameshift mutation affecting herpes simplex virus thymidine kinase is sufficient to permit reactivation from latency. J Virol 77: 4703–4709. 12663777 10.1128/JVI.77.8.4703-4709.2003 PMC152167

[10] Griffiths A, Coen DM (2003) High-frequency phenotypic reversion and pathogenicity of an acyclovir-resistant herpes simplex virus mutant. J Virol 77: 2282–2286. 12525666 10.1128/JVI.77.3.2282-2286.2003 PMC140925

[11] Plummer G, Goodheart CR, Miyagi M, Skinner GR, Thouless ME, et al (1974) Herpes simplex viruses: discrimination of types and correlation between different characteristics. Virology 60: 206–216. 4366799 10.1016/0042-6822(74)90378-x

[12] Lopez C (1975) Genetics of natural resistance to herpesvirus infections in mice. Nature 258: 152–153. 171586 10.1038/258152a0

[13] Steiner I, Benninger F (2013) Update on herpes virus infections of the nervous system. Current neurology and neuroscience reports 13: 414. 24142852 10.1007/s11910-013-0414-8

[14] Metcalf JF, Michaelis BA (1984) Herpetic keratitis in inbred mice. Invest Ophthalmol Vis Sci 25: 1222–1225. 6480298

[15] Hazlett LD, Hendricks RL (2010) Reviews for immune privilege in the year 2010: immune privilege and infection. Ocular immunology and inflammation 18: 237–243. 20662654 10.3109/09273948.2010.501946

[16] Gimenez F, Suryawanshi A, Rouse BT (2013) Pathogenesis of herpes stromal keratitis—a focus on corneal neovascularization. Prog Retin Eye Res 33: 1–9. 22892644 10.1016/j.preteyeres.2012.07.002 PMC3511644

[17] Veiga-Parga T, Suryawanshi A, Mulik S, Gimenez F, Sharma S, et al (2012) On the role of regulatory T cells during viral-induced inflammatory lesions. J Immunol 189: 5924–5933. 23129753 10.4049/jimmunol.1202322 PMC3518750

[18] Foster CS, Tsai Y, Monroe JG, Campbell R, Cestari M, et al (1986) Genetic studies on murine susceptibility to herpes simplex keratitis. Clin Immunol Immunopathol 40: 313–325. 3013475 10.1016/0090-1229(86)90036-x

[19] Lundberg P, Welander P, Openshaw H, Nalbandian C, Edwards C, et al (2003) A locus on mouse chromosome 6 that determines resistance to herpes simplex virus also influences reactivation, while an unlinked locus augments resistance of female mice. J Virol 77: 11661–11673. 14557652 10.1128/JVI.77.21.11661-11673.2003 PMC229335

[20] Kriesel JD, Jones BB, Matsunami N, Patel MK, St Pierre CA, et al (2011) C21orf91 genotypes correlate with herpes simplex labialis (cold sore) frequency: description of a cold sore susceptibility gene. J Infect Dis 204: 1654–1662. 22039568 10.1093/infdis/jir633 PMC3203230

[21] Hobbs MR, Jones BB, Otterud BE, Leppert M, Kriesel JD (2008) Identification of a herpes simplex labialis susceptibility region on human chromosome 21. J Infect Dis 197: 340–346. 18199027 10.1086/525540

[22] Peirce JL, Lu L, Gu J, Silver LM, Williams RW (2004) A new set of BXD recombinant inbred lines from advanced intercross populations in mice. BMC Genet 5: 7. 15117419 10.1186/1471-2156-5-7 PMC420238

[23] Lu H, Li L, Watson ER, Williams RW, Geisert EE, et al (2011) Complex interactions of Tyrp1 in the eye. Mol Vis 17: 2455–2468. 21976956 PMC3185026

[24] Geisert EE, Lu L, Freeman-Anderson NE, Templeton JP, Nassr M, et al (2009) Gene expression in the mouse eye: an online resource for genetics using 103 strains of mice. Mol Vis 15: 1730–1763. 19727342 PMC2736153

[25] Carneiro AM, Airey DC, Thompson B, Zhu CB, Lu L, et al (2009) Functional coding variation in recombinant inbred mouse lines reveals multiple serotonin transporter-associated phenotypes. Proc Natl Acad Sci U S A 106: 2047–2052. 19179283 10.1073/pnas.0809449106 PMC2632716

[26] Rulten SL, Ripley TL, Hunt CL, Stephens DN, Mayne LV (2006) Sp1 and NFkappaB pathways are regulated in brain in response to acute and chronic ethanol. Genes Brain Behav 5: 257–273. 16594979 10.1111/j.1601-183X.2005.00157.x

[27] Alexander RC, Wright R, Freed W (1996) Quantitative trait loci contributing to phencyclidine-induced and amphetamine-induced locomotor behavior in inbred mice. Neuropsychopharmacology 15: 484–490. 8914121 10.1016/S0893-133X(96)00058-9

[28] Aziz RK, Kansal R, Abdeltawab NF, Rowe SL, Su Y, et al (2007) Susceptibility to severe Streptococcal sepsis: use of a large set of isogenic mouse lines to study genetic and environmental factors. Genes Immun 8: 404–415. 17525705 10.1038/sj.gene.6364402

[29] Zumbrun EE, Abdeltawab NF, Bloomfield HA, Chance TB, Nichols DK, et al (2011) Development of a murine model for aerosolized ebolavirus infection using a panel of recombinant inbred mice. Viruses 4: 3468–3493. 10.3390/v4123468 PMC3528275 23207275

[30] Nedelko T, Kollmus H, Klawonn F, Spijker S, Lu L, et al (2012) Distinct gene loci control the host response to influenza H1N1 virus infection in a time-dependent manner. BMC Genomics 13: 411. 22905720 10.1186/1471-2164-13-411 PMC3479429

[31] Yadav JS, Pradhan S, Kapoor R, Bangar H, Burzynski BB, et al (2011) Multigenic control and sex bias in host susceptibility to spore-induced pulmonary anthrax in mice. Infect Immun 79: 3204–3215. 21628518 10.1128/IAI.01389-10 PMC3147590

[32] Sawtell NM, Thompson RL (2004) Comparison of herpes simplex virus reactivation in ganglia in vivo and in explants demonstrates quantitative and qualitative differences. J Virol 78: 7784–7794. 15220452 10.1128/JVI.78.14.7784-7794.2004 PMC434126

[33] Thompson RL, Preston CM, Sawtell NM (2009) De novo synthesis of VP16 coordinates the exit from HSV latency in vivo. PLoS Pathog 5: e1000352. 19325890 10.1371/journal.ppat.1000352 PMC2654966

[34] Thompson RL, Sawtell NM (2011) The herpes simplex virus type 1 latency associated transcript locus is required for the maintenance of reactivation competent latent infections. J Neurovirol 17: 552–558. 22207584 10.1007/s13365-011-0071-0 PMC3299008

[35] Brandt CR (2004) Virulence genes in herpes simplex virus type 1 corneal infection. Curr Eye Res 29: 103–117. 15512957 10.1080/02713680490504533

[36] Brandt CR (2005) The role of viral and host genes in corneal infection with herpes simplex virus type 1. Exp Eye Res 80: 607–621. 15862167 10.1016/j.exer.2004.09.007

[37] Brandt CR, Coakley LM, Grau DR (1992) A murine model of herpes simplex virus-induced ocular disease for antiviral drug testing. J Virol Methods 36: 209–222. 1560105 10.1016/0166-0934(92)90052-f

[38] Shimeld C, Tullo AB, Hill TJ, Blyth WA, Easty DL (1985) Spread of herpes simplex virus and distribution of latent infection after intraocular infection of the mouse. Archives of virology 85: 175–187. 2992417 10.1007/BF01314229

[39] Whitley RJ (2001) Chapter 73: Herpes Simplex Viruses. In: P. M. H. David M Knipe, editor editors. Field's Virology. Lippincott Williams & Wilkins. pp. 2461–2510.

[40] Thompson RL, Stevens JG (1983) Biological characterization of a herpes simplex virus intertypic recombinant which is completely and specifically non-neurovirulent. Virology 131: 171–179. 6316649 10.1016/0042-6822(83)90543-3

[41] Thompson RL, Cook ML, Devi-Rao GB, Wagner EK, Stevens JG (1986) Functional and molecular analyses of the avirulent wild-type herpes simplex virus type 1 strain KOS. J Virol 58: 203–211. 3005649 10.1128/jvi.58.1.203-211.1986 PMC252894

[42] Thompson RL, Rogers SK, Zerhusen MA (1989) Herpes simplex virus neurovirulence and productive infection of neural cells is associated with a function which maps between 0.82 and 0.832 map units on the HSV genome. Virology 172: 435–450. 2552657 10.1016/0042-6822(89)90186-4

[43] Hill TJ, Yirrell DL, Blyth WA (1986) Infection of the adrenal gland as a route to the central nervous system after viraemia with herpes simplex virus in the mouse. J Gen Virol 67 ( Pt 2): 309–320. 10.1099/0022-1317-67-2-309 3003239

[44] Han X, Lundberg P, Tanamachi B, Openshaw H, Longmate J, et al (2001) Gender influences herpes simplex virus type 1 infection in normal and gamma interferon-mutant mice. J Virol 75: 3048–3052. 11222734 10.1128/JVI.75.6.3048-3052.2001 PMC115935

[45] Broman KW (2001) Review of statistical methods for QTL mapping in experimental crosses. Lab Anim (NY) 30: 44–52. 11469113

[46] Brandt CR, Akkarawongsa R, Altmann S, Jose G, Kolb AW, et al (2007) Evaluation of a theta-defensin in a Murine model of herpes simplex virus type 1 keratitis. Invest Ophthalmol Vis Sci 48: 5118–5124. 17962464 10.1167/iovs.07-0302

[47] Zhao ZS, Granucci F, Yeh L, Schaffer PA, Cantor H (1998) Molecular mimicry by herpes simplex virus-type 1: autoimmune disease after viral infection. Science 279: 1344–1347. 9478893 10.1126/science.279.5355.1344

[48] Suryawanshi A, Veiga-Parga T, Rajasagi NK, Reddy PB, Sehrawat S, et al (2011) Role of IL-17 and Th17 cells in herpes simplex virus-induced corneal immunopathology. J Immunol 187: 1919–1930. 21765013 10.4049/jimmunol.1100736 PMC3150378

[49] Veiga-Parga T, Gimenez F, Mulik S, Chiang EY, Grogan JL, et al (2012) Controlling herpetic stromal keratitis by modulating lymphotoxin-alpha-mediated inflammatory pathways. Microbes and infection / Institut Pasteur 15: 677–687. 10.1016/j.micinf.2013.07.001 PMC3769451 23850656

[50] Inoue Y (2008) Immunological aspects of herpetic stromal keratitis. Semin Ophthalmol 23: 221–227. 18584559 10.1080/08820530802111390

[51] Taylor BA, Heiniger HJ, Meier H (1973) Genetic analysis of resistance to cadmium-induced testicular damage in mice. Proc Soc Exp Biol Med 143: 629–633. 4719448 10.3181/00379727-143-37380

[52] Norose K, Yano A, Zhang XM, Blankenhorn E, Heber-Katz E (2002) Mapping of genes involved in murine herpes simplex virus keratitis: identification of genes and their modifiers. J Virol 76: 3502–3510. 11884574 10.1128/JVI.76.7.3502-3510.2002 PMC136007

[53] Huster KM, Panoutsakopoulou V, Prince K, Sanchirico ME, Cantor H (2002) T cell-dependent and -independent pathways to tissue destruction following herpes simplex virus-1 infection. Eur J Immunol 32: 1414–1419. 11981829 10.1002/1521-4141(200205)32:5<1414::AID-IMMU1414>3.0.CO;2-Q

[54] Gu W, Huang Q, Hayward GS (1995) Multiple Tandemly Repeated Binding Sites for the YY1 Repressor and Transcription Factors AP-1 and SP-1 Are Clustered within Intron-1 of the Gene Encoding the IE110 Transactivator of Herpes simplex Virus Type 1. J Biomed Sci 2: 203–226. 11725057 10.1007/BF02253381

[55] Lieu PT, Wagner EK (2000) Two leaky-late HSV-1 promoters differ significantly in structural architecture. Virology 272: 191–203. 10873762 10.1006/viro.2000.0365

[56] Overall RW, Kempermann G, Peirce J, Lu L, Goldowitz D, et al (2009) Genetics of the hippocampal transcriptome in mouse: a systematic survey and online neurogenomics resource. Front Neurosci 3: 55. 20582282 10.3389/neuro.15.003.2009 PMC2858614

[57] Roizman B, Gu H, Mandel G (2005) The first 30 minutes in the life of a virus: unREST in the nucleus. Cell Cycle 4: 1019–1021. 16082207 10.4161/cc.4.8.1902

[58] Du T, Zhou G, Khan S, Gu H, Roizman B (2010) Disruption of HDAC/CoREST/REST repressor by dnREST reduces genome silencing and increases virulence of herpes simplex virus. Proc Natl Acad Sci U S A 107: 15904–15909. 20798038 10.1073/pnas.1010741107 PMC2936609

[59] Guo Y, Audry M, Ciancanelli M, Alsina L, Azevedo J, et al (2011) Herpes simplex virus encephalitis in a patient with complete TLR3 deficiency: TLR3 is otherwise redundant in protective immunity. J Exp Med 208: 2083–2098. 21911422 10.1084/jem.20101568 PMC3182056

[60] Sancho-Shimizu V, Perez de Diego R, Lorenzo L, Halwani R, Alangari A, et al (2011) Herpes simplex encephalitis in children with autosomal recessive and dominant TRIF deficiency. J Clin Invest 121: 4889–4902. 22105173 10.1172/JCI59259 PMC3226004

[61] Zhang SY, Abel L, Casanova JL (2013) Mendelian predisposition to herpes simplex encephalitis. Handb Clin Neurol 112: 1091–1097. 23622315 10.1016/B978-0-444-52910-7.00027-1

[62] Cantin E, Tanamachi B, Openshaw H (1999) Role for gamma interferon in control of herpes simplex virus type 1 reactivation. J Virol 73: 3418–3423. 10074196 10.1128/jvi.73.4.3418-3423.1999 PMC104106

[63] Cantin E, Tanamachi B, Openshaw H, Mann J, Clarke K (1999) Gamma interferon (IFN-gamma) receptor null-mutant mice are more susceptible to herpes simplex virus type 1 infection than IFN-gamma ligand null-mutant mice. J Virol 73: 5196–5200. 10233988 10.1128/jvi.73.6.5196-5200.1999 PMC112570

[64] Leib DA, Harrison TE, Laslo KM, Machalek MA, Moorman NJ, et al (1999) Interferons regulate the phenotype of wild-type and mutant herpes simplex viruses in vivo. J Exp Med 189: 663–672. 9989981 10.1084/jem.189.4.663 PMC2192939

[65] Everett RD, Boutell C, McNair C, Grant L, Orr A (2010) Comparison of the biological and biochemical activities of several members of the alphaherpesvirus ICP0 family of proteins. J Virol 84: 3476–3487. 20106921 10.1128/JVI.02544-09 PMC2838103

[66] Halford WP, Weisend C, Grace J, Soboleski M, Carr DJ, et al (2006) ICP0 antagonizes Stat 1-dependent repression of herpes simplex virus: implications for the regulation of viral latency. Virol J 3: 44. 16764725 10.1186/1743-422X-3-44 PMC1557838

[67] Pruett ND, Tkatchenko TV, Jave-Suarez L, Jacobs DF, Potter CS, et al (2004) Krtap16, characterization of a new hair keratin-associated protein (KAP) gene complex on mouse chromosome 16 and evidence for regulation by Hoxc13. J Biol Chem 279: 51524–51533. 15385554 10.1074/jbc.M404331200

[68] Bourane S, Mechaly I, Venteo S, Garces A, Fichard A, et al (2007) A SAGE-based screen for genes expressed in sub-populations of neurons in the mouse dorsal root ganglion. BMC Neurosci 8: 97. 18021428 10.1186/1471-2202-8-97 PMC2241628

[69] Piers TM, Kim DH, Kim BC, Regan P, Whitcomb DJ, et al (2012) Translational Concepts of mGluR5 in Synaptic Diseases of the Brain. Front Pharmacol 3: 199. 23205012 10.3389/fphar.2012.00199 PMC3506921

[70] Conejero-Goldberg C, Torrey EF, Yolken RH (2003) Herpesviruses and Toxoplasma gondii in orbital frontal cortex of psychiatric patients. Schizophr Res 60: 65–69. 12505139 10.1016/s0920-9964(02)00160-3

[71] Rantakallio P, Jones P, Moring J, Von Wendt L (1997) Association between central nervous system infections during childhood and adult onset schizophrenia and other psychoses: a 28-year follow-up. Int J Epidemiol 26: 837–843. 9279617 10.1093/ije/26.4.837

[72] Kanno H, Ozawa H, Dohi Y, Sekiguchi A, Igarashi K, et al (2009) Genetic ablation of transcription repressor Bach1 reduces neural tissue damage and improves locomotor function after spinal cord injury in mice. J Neurotrauma 26: 31–39. 19119918 10.1089/neu.2008.0667

[73] Chu J, Hong NA, Masuda CA, Jenkins BV, Nelms KA, et al (2009) A mouse forward genetics screen identifies LISTERIN as an E3 ubiquitin ligase involved in neurodegeneration. Proc Natl Acad Sci U S A 106: 2097–2103. 19196968 10.1073/pnas.0812819106 PMC2650114

[74] Kriesel JD, Gebhardt BM, Hill JM, Maulden SA, Hwang IP, et al (1997) Anti-interleukin-6 antibodies inhibit herpes simplex virus reactivation. J Infect Dis 175: 821–827. 9086136 10.1086/513977

