# Supplementary material for: A Forward Phenotypically Driven Unbiased Genetic Analysis of Host Genes That Moderate Herpes Simplex Virus Virulence and Stromal Keratitis in Mice
Source: PLoS One. 2014 Mar 20;9(3):e92342. doi: 10.1371/journal.pone.0092342 (PMC3961320; doi:10.1371/journal.pone.0092342)
Supplement: Table S1 — Shown are the known and predicted genes under and near the Chr 16 QTL for percent mortality detailed in figure 7 . Genes within the QTL are highlited in light blue (entries 7 to 70). An interactive table containing more information can be generated at www.genenetwork.org by using the GeneNetwork Interval Analyst for Chr 16 gated between 85 and 90 Mb. (DOCX) [file pone.0092342.s001.docx]

Table S1

| **Column1** | | **Column2** | | **Column3** | **Column4** | **Column5** |
| --- | --- | --- | --- | --- | --- | --- |
| GeneNetwork Interval Analyst Table | |  | |  |  |  |
| <http://132.192.47.32/webqtl/main.py?FormID=intervalAnalyst> | | | | |  |  |
| Date : May 31, 2013 | | | |  |  |  |
| Time : 17:40 GMT | | | |  |  |  |
| Search by : XXX.XXX.XXX.XXX | | | | |  |  |
| view region : Chr 16 85.000000 - 90.000000 Mb | | | | |  |  |
|  |  | |  | |  |  |
| **Index** | **Gene** | | **Description (mm9)** | | **Chr (mm9)** | **Start (mm9)** |
| 1 | App | | amyloid beta (A4) precursor protein | | 16 | 84.954687 |
| 2 | Cyyr1 | | cysteine and tyrosine-rich protein 1 | | 16 | 85.456489 |
| 3 | Adamts1 | | a disintegrin-like and metallopeptidase (reprolysin type) with thrombospondin type 1 motif, 1 | | 16 | 85.794072 |
| 4 | 4930556C24Rik | | RIKEN cDNA 4930556C24 gene | | 16 | 85.819455 |
| 5 | Adamts5 | | a disintegrin-like and metallopeptidase (reprolysin type) with thrombospondin type 1 motif, 5 (aggrecanase-2) | | 16 | 85.858401 |
| 6 | 1700007H22Rik | | RIKEN cDNA 1700007H22 gene | | 16 | 86.139548 |
| 7 | EG665393 | | predicted gene, EG665393 | | 16 | 86.664094 |
| 8 | LOC100040088 | | hypothetical protein LOC100040088 | | 16 | 87.328128 |
| 9 | N6amt1 | | N-6 adenine-specific DNA methyltransferase 1 (putative) | | 16 | 87.354429 |
| 10 | Zfp294 | | zinc finger protein 294 (embyonic neurodegeneration) | | 16 | 87.376895 |
| 11 | Rwdd2b | | RWD domain containing 2B | | 16 | 87.433575 |
| 12 | Usp16 | | ubiquitin specific peptidase 16 | | 16 | 87.4553 |
| 13 | Cct8 | | chaperonin subunit 8 (theta) | | 16 | 87.483569 |
| 14 | B130034C11Rik | | RIKEN cDNA B130034C11 gene | | 16 | 87.496317 |
| 15 | ORF63 | | open reading frame 63 | | 16 | 87.553574 |
| 16 | Bach1 | | BTB and CNC homology 1 (ubiquitously expressed) | | 16 | 87.699198 |
| 17 | 2810407A14Rik | | RIKEN cDNA 2810407A14 gene | | 16 | 87.784319 |
| 18 | Grik1 | | glutamate receptor, ionotropic, kainate 1 | | 16 | 87.896141 |
| 19 | 4930590A17Rik | | RIKEN cDNA 4930590A17 gene | | 16 | 87.909515 |
| 20 | 4930420G21Rik | | RIKEN cDNA 4930420G21 gene | | 16 | 88.42662 |
| 21 | Cldn17 | | claudin 17 | | 16 | 88.506051 |
| 22 | Cldn8 | | claudin 8 | | 16 | 88.56107 |
| 23 | Gm312 | | gene model 312, (NCBI) | | 16 | 88.610953 |
| 24 | 2310079G19Rik | | RIKEN cDNA 2310079G19 gene | | 16 | 88.627032 |
| 25 | Krtap26-1 | | keratin associated protein 26-1 | | 16 | 88.647068 |
| 26 | 2310061N02Rik | | RIKEN cDNA 2310061N02 gene | | 16 | 88.707415 |
| 27 | Krtap13-1 | | keratin associated protein 13-1 | | 16 | 88.729106 |
| 28 | Krtap13 | | keratin associated protein 13 | | 16 | 88.750989 |
| 29 | Krtap16-2 | | keratin associated protein 16-2 | | 16 | 88.757922 |
| 30 | 2310034C09Rik | | RIKEN cDNA 2310034C09 gene | | 16 | 88.759091 |
| 31 | 2310057N15Rik | | RIKEN cDNA 2310057N15 gene | | 16 | 88.773425 |
| 32 | 4930553J12Rik | | RIKEN cDNA 4930553J12 gene | | 16 | 88.799136 |
| 33 | EG433047 | | predicted gene, EG433047 | | 16 | 88.811685 |
| 34 | Krtap14 | | keratin associated protein 14 | | 16 | 88.825535 |
| 35 | Krtap15 | | keratin associated protein 15 | | 16 | 88.829253 |
| 36 | Krtap16-9 | | keratin associated protein 16-9 | | 16 | 88.869162 |
| 37 | Krtap16-6 | | keratin associated protein 16-6 | | 16 | 88.869367 |
| 38 | Krtap16-1 | | keratin associated protein 16-1 | | 16 | 88.873909 |
| 39 | Krtap16-5 | | keratin associated protein 16-5 | | 16 | 88.877757 |
| 40 | Krtap16-4 | | keratin associated protein 16-4 | | 16 | 88.88503 |
| 41 | Krtap8-2 | | keratin associated protein 8-2 | | 16 | 88.896212 |
| 42 | AY026312 | | cDNA sequence AY026312 | | 16 | 88.932048 |
| 43 | Krtap16-10 | | keratin associated protein 16-10 | | 16 | 88.962548 |
| 44 | Krtap16-3 | | keratin associated protein 16-3 | | 16 | 88.962551 |
| 45 | 1110032D16Rik | | RIKEN cDNA 1110032D16 gene | | 16 | 89.010639 |
| 46 | Krtap6-1 | | keratin associated protein 6-1 | | 16 | 89.031943 |
| 47 | ENSMUSG00000068 | | predicted gene, ENSMUSG00000068790 | | 16 | 89.041167 |
| 48 | ENSMUSG00000068 | | predicted gene, ENSMUSG00000068396 | | 16 | 89.041167 |
| 49 | ENSMUSG00000068 | | predicted gene, ENSMUSG00000068601 | | 16 | 89.041167 |
| 50 | ENSMUSG00000068 | | predicted gene, ENSMUSG00000068924 | | 16 | 89.041167 |
| 51 | ENSMUSG00000068 | | predicted gene, ENSMUSG00000068680 | | 16 | 89.041167 |
| 52 | ENSMUSG00000068 | | predicted gene, ENSMUSG00000068623 | | 16 | 89.041167 |
| 53 | ENSMUSG00000068 | | predicted gene, ENSMUSG00000068293 | | 16 | 89.041167 |
| 54 | ENSMUSG00000068 | | predicted gene, ENSMUSG00000068279 | | 16 | 89.041167 |
| 55 | ENSMUSG00000068 | | predicted gene, ENSMUSG00000068074 | | 16 | 89.041167 |
| 56 | ENSMUSG00000068 | | predicted gene, ENSMUSG00000068910 | | 16 | 89.041167 |
| 57 | ENSMUSG00000068 | | predicted gene, ENSMUSG00000068179 | | 16 | 89.041167 |
| 58 | ENSMUSG00000068 | | predicted gene, ENSMUSG00000068075 | | 16 | 89.041167 |
| 59 | Krtap16-8 | | keratin associated protein 16-8 | | 16 | 89.047533 |
| 60 | 1110025L11Rik | | RIKEN cDNA 1110025L11 gene | | 16 | 89.063658 |
| 61 | Krtap6-3 | | keratin associated protein 6-3 | | 16 | 89.084335 |
| 62 | ENSMUSG00000044 | | predicted gene, ENSMUSG00000044227 | | 16 | 89.15816 |
| 63 | 1110057P08Rik | | RIKEN cDNA 1110057P08 gene | | 16 | 89.174979 |
| 64 | Krtap20-2 | | keratin associated protein 20-2 | | 16 | 89.206105 |
| 65 | Krtap8-1 | | keratin associated protein 8-1 | | 16 | 89.376258 |
| 66 | Krtap16-7 | | keratin associated protein 16-7 | | 16 | 89.403271 |
| 67 | Krtap6-2 | | keratin associated protein 6-2 | | 16 | 89.419567 |
| 68 | Krtap7-1 | | keratin associated protein 7-1 | | 16 | 89.507948 |
| 69 | Krtap11-1 | | keratin associated protein 11-1 | | 16 | 89.57042 |
| 70 | Tiam1 | | T-cell lymphoma invasion and metastasis 1 | | 16 | 89.787355 |

Shown are the known and predicted genes under and near the Chr 16 QTL for percent mortality detailed in figure 7. Genes within the QTL are highlited in light blue (entries 7 to 70). An interactive table containing more information can be generated at [www.genenetwork.org](http://www.genenetwork.org) by using the GeneNetwork Interval Analyst for Chr 16 gated between 85 and 90 Mb.
